# Cell Based Technologies: Abstracts of the 22^nd^ ESACT Meeting 2011 in Vienna

**DOI:** 10.1186/1753-6561-5-S8-I1

**Published:** 2011-11-22

**Authors:** Hansjörg Hauser

**Affiliations:** 1 Helmholtz-Zentrum für Infektionsforschung GmbH, Department of Gene Regulation and Differentiation, 38124 Braunschweig, Germany

## 

The European Society of Animal Cell Technology (ESACT) is a society that brings together scientists, engineers and other specialists working with animal cells in order to promote communication of experiences between European and international investigators and progress development of cell systems in productions derived from them.

Animal cells are being used as substrates in basic research and also for the production of proteins. Tissue engineering, gene and cell therapies, organ replacement technologies and cell-based biosensors contribute to a considerable widening of interest and research activity based on animal cell technology.

The abstracts of this supplement are from the 22^nd^ ESACT meeting that was held in Vienna, Austria, May 15-18, 2011. The abstracts review the presentations from this meeting and should be a useful resource for the state-of-the-art in animal cell technology.

